# Vagus Nerve Mediated Liver-Brain-Axis Is a Major Regulator of the Metabolic Landscape in the Liver

**DOI:** 10.3390/ijms26052166

**Published:** 2025-02-28

**Authors:** Camila F. Brito, Roberta C. Fonseca, Lucas Rodrigues-Ribeiro, João S. F. Guimarães, Bruna F. Vaz, Gabriel S. S. Tofani, Ana C. S. Batista, Ariane B. Diniz, Paola Fernandes, Núbia A. M. Nunes, Rafaela M. Pessoa, Amanda C. C. Oliveira, Ivana S. Lula, Valbert N. Cardoso, Simone O. A. Fernandes, Maristela O. Poletini, Jacqueline I. Alvarez-Leite, Gustavo B. Menezes, Adaliene V. M. Ferreira, Mariana T. Q. Magalhães, Vladimir Gorshkov, Frank Kjeldsen, Thiago Verano-Braga, Alan M. Araujo, André G. Oliveira

**Affiliations:** 1 Department of Physiology and Biophysics, Institute of Biological Sciences, Universidade Federal de Minas Gerais, Belo Horizonte 31270-901, Brazil; camilabrito641@gmail.com (C.F.B.); rcristellif@gmail.com (R.C.F.);; 2 Department of Biochemistry and Immunology, Institute of Biological Sciences, Universidade Federal de Minas Gerais, Belo Horizonte 31270-901, Brazil; 3 Department of Morphology, Institute of Biological Sciences, Universidade Federal de Minas Gerais, Belo Horizonte 31270-901, Brazil; 4 Department of Clinical and Toxicological Analysis, College of Pharmacy, Universidade Federal de Minas Gerais, Belo Horizonte 31270-901, Brazil; 5 Department of Nutrition, Nursing School, Universidade Federal de Minas Gerais, Belo Horizonte 31270-901, Brazil; 6 Department of Chemistry, Universidade Federal de Minas Gerais, Belo Horizonte 31270-901, Brazil; ivanalula@ufmg.br; 7 Department of Biochemistry and Molecular Biology, University of Southern Denmark, 5230 Odense, Denmark; 8 Department of Neuroscience, Perelman School of Medicine, University of Pennsylvania, Philadelphia, PA 19104, USA; 9 Monell Chemical Senses Center, Philadelphia, PA 19104, USA

**Keywords:** liver-brain axis, vagus nerve, metabolism, steatosis

## Abstract

The liver serves as a major energetic reservoir for other tissues and its metabolic function is controlled by humoral and neural factors. The vagus nerve innervating the gastrointestinal tract plays an important role in regulating peripheral metabolism and energy expenditure. Although the liver receives vagus nerve fibers, the impact of this circuitry in the regulation of hepatic metabolism is still poorly understood. Herein, we used a combination of quantitative proteomics and in vivo imaging techniques to investigate the impact of the vagus nerve on liver metabolism in male mice. Liver-brain axis was impaired by vagotomy (VNX) or knocking down of the vesicular acetylcholine transporter (VAChT-KD). Mice were challenged with high carbohydrate or high-fat feeding. The vagus nerve shapes the metabolic framework of the liver, as vagotomy led to a significant alteration of the hepatic proteome landscape. Differential protein expression and pathway enrichment analyses showed that glycolytic and fatty acid biosynthesis were increased following VNX, whereas β-oxidation was decreased. These results were corroborated in VAChT-KD mice. This metabolic shift facilitated lipid accumulation in hepatocytes in mice fed with a standard commercial diet. Furthermore, VNX worsened liver steatosis following high-carbohydrate or high-fat dietary challenges. This study describes the liver-brain axis mediated by the vagus nerve as an important regulator of the hepatic metabolic landscape.

## 1. Introduction

Heterotrophic organisms depend on nutrients to fuel metabolism and support life [1]. Following digestion and absorption in the intestines, nutrients, and microbiota-derived metabolites enter the liver, a central metabolic hub in the organism, through the hepatic portal circulation.

The hepatic parenchyma is a highly specialized microenvironment that supports and integrates several key metabolic frameworks and reactions [2] that are important to produce and store the energy used to sustain physiological functions at the cellular, tissue, and organ levels [3]. Liver metabolism is highly dynamic and is regulated by nutrient availability and humoral factors, which confer the flexibility needed for adaptation to fed and fasting states. In addition, a growing body of evidence suggests that the central autonomic nervous system also plays an important role in coordinating hepatic metabolic functions [4].

The vagus nerve is an important source of autonomic innervation in the gastrointestinal tract [5]. Because it contains both afferent and efferent fibers, the vagus nerve conveys a bidirectional circuitry bridging these organs and the brain. Notably, a great number of vagal fibers derived from the hepatic branch innervate the liver and thus may couple nutrient ingestion with metabolism. The concept is that vagal afferent innervation senses mechanical distention of the digestive tube following food ingestion, paralleled by a vagal-mediated neuroepithelial circuit that senses nutrients within the intestinal lumen [6,7]. In addition, a role for hepatic vagal innervation in nutrient sensing has been proposed, as the portal infusion of glucose, amino acids, and lipids is shown to increase hepatic vagal activity [8,9,10,11]. These stimuli are integrated in different brain areas and are classically involved in learning food preferences, controlling meal size (satiation), as well as proper digestion and absorption of nutrients [12,13]. Not surprisingly, the disruption in the neurophysiology of the gut-brain axis has been associated with metabolic diseases. In this regard, the reduction in vagus nerve sensitivity or plasticity resulted in hyperphagia and led to obesity [14]. In line with these findings, electrical stimulation of the vagus nerve reduced food intake, body weight/fat, and increased energy expenditure [15,16,17].

Here, we show that in addition to sensing mechanical and nutrient stimuli, the vagus nerve displays an important role in shaping the metabolic framework of the liver. Furthermore, we show that the disruption in this circuitry shifts hepatic metabolism and facilitates lipid accumulation in hepatocytes.

## 2. Results

### 2.1. Vagotomy Leads to Hepatic Proteome Remodeling

To elucidate the processes regulated by the vagus nerve in the liver, we first performed quantitative proteomics that allowed a comparison of protein-relative expressions between vagotomized mice (VNX) and sham-operated control animals (Sham). In total, our proteomic analysis of five biological samples for each group allowed the identification and quantification of 2121 proteins (Supplementary Table S1). Importantly, aiming to increase the robustness of downstream analysis, we did not exclude any biological replication. Furthermore, if a certain protein was not identified in all samples, it was not included in our study.

**Figure 1:**
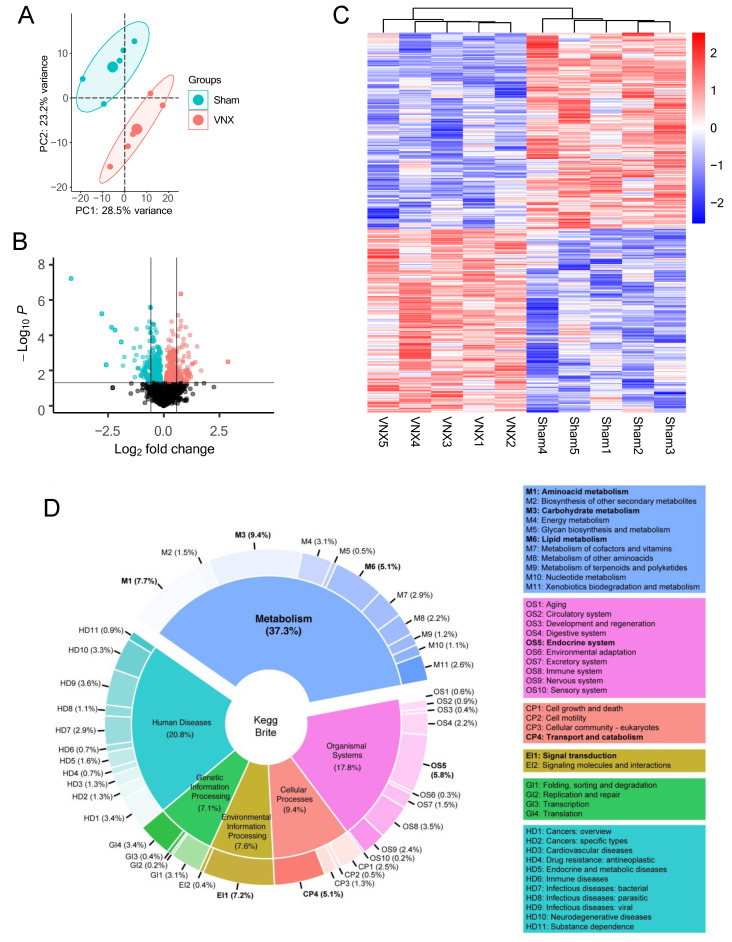
Liver proteome remodeling following vagotomy. (**A**) PCA of relative protein expression in VNX and Sham animals. (**B**) Volcano plot of protein expression. Blue and red colors respectively represent statistically decreased and increased expression in VNX mice compared to Sham animals (n = 5/group; *p* < 0.05, FDR adjusted-*p* < 0.05). (**C**) Significantly regulated protein patterns in individual samples. (**D**) Summary of significantly regulated proteins mapped onto the KEGG database. VNX = vagotomy.

Principal component analysis (PCA) showed that Sham and VNX animals had distinctly different proteomes, as both groups clustered apart (Figure 1A). We also identified 453 differentially expressed proteins between VNX and Sham groups, corresponding to approximately 21% of the total proteins, of which 235 were downregulated and 219 upregulated following vagotomy (Figure 1B,C). Taken together, our results indicate that the proteome of the liver was remodeled following vagotomy.

### 2.2. Metabolism Is a Major Target of the Vagal Neural Circuit in the Liver

To gain insights into the biological functions associated with the differentially abundant proteins following VNX, we performed an exploratory analysis using the hierarchical context of the KEGG Brite database. In the first Brite level, these proteins covered 37.3% of biological objects classified within the “Metabolism” category, followed by objects categorized into “Human Diseases” (20.8%), “Organismal Systems” (17.8%), “Cellular Processes” (9.4%), “Environmental Information Processing” (7.6%), and “Genetic Information Processing” (7.1%) (Figure 1D). In a further classification level, we observed that the different abundant proteins covered functions classified into key metabolic and/or regulatory processes, including “Carbohydrate metabolism” (9.4%), “Amino acid metabolism” (7.7%), “Lipid metabolism” (5.1%), “Energy metabolism” (3.1%), Endocrine system (5.8%), and “Signal transduction” (7.2%) (Figure 1D).

**Figure 2:**
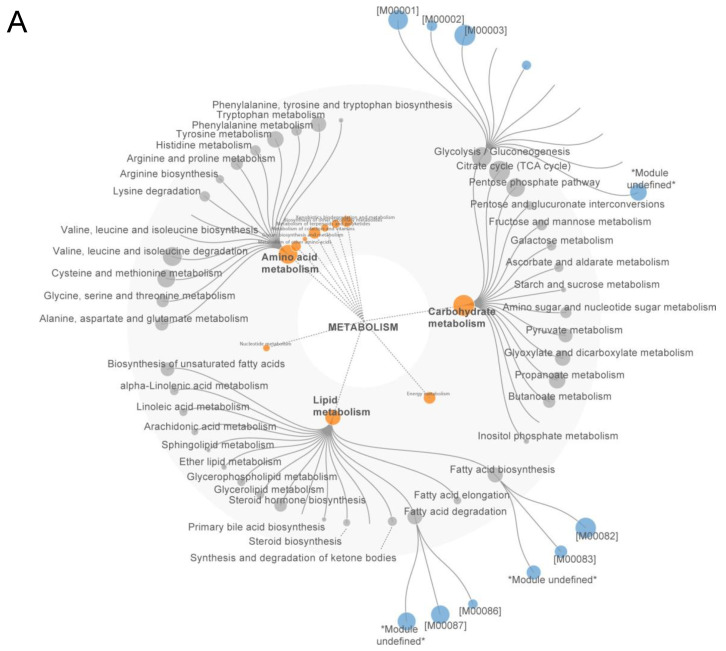

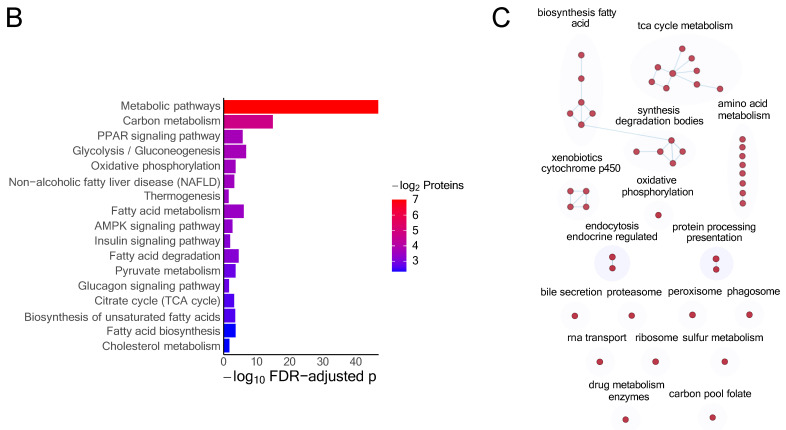
Functional enrichment analysis reveals metabolism as a major target of vagus nerve circuitry. (**A**) Functional mapping, (**B**) enrichment analysis, and (**C**) pathway clustering network of differentially abundant proteins following vagotomy. Node size in (**A**) corresponds to the inverse FDR-adjusted *p*-values. M00001 = Glycolysis (Embden-Meyerhof pathway), glucose => pyruvate; M00002 = Glycolysis, core module involving three-carbon compounds; M00003 = Gluconeogenesis, oxaloacetate => fructose-6P; M00082 = Fatty acid biosynthesis, initiation; M00083 = Fatty acid biosynthesis, elongation; M00086 = β-Oxidation, acyl-CoA synthesis; M00087 = β-Oxidation. In (**B**), enriched pathways were considered when FDR < 0.05. Node in (**C**) represents enriched pathways and text corresponds to the word count of terms associated with the pathways within each cluster.

Next, we mapped the proteins onto a hierarchical dendrogram that comprehends all known biological functions of the KEGG database, where each layer of nodes is mapped to a level of hierarchy. In addition, we also mapped the sum of the inverse *p*-value to the radius of each node, so that the node size could facilitate the visualization of putative functional clusters altered by vagotomy. Based on our previous results, we set the root of the dendrogram in Metabolism, as it was the Brite category with more coverage. We confirmed that carbohydrate, lipid, and amino acid metabolism were the main metabolic processes impacted by vagotomy (Figure 2A). This analysis also allowed the investigation of biological pathways within these categories and revealed that core metabolic pathways and subpathways (or modules) in the liver depend on the proteins regulated by the vagus nerve. In this sense, we observed that glycolysis, including Embden- Meyerhof pathway (glycolysis => pyruvate and compounds); gluconeogenesis (oxaloacetate => fructose-6P); fatty acid biosynthesis (initiation and elongation) and fatty acid degradation (β-oxidation) were important targets regulated by the vagus nerve and that VNX potentially disrupted the hepatic metabolic flux (Figure 2A).

Not surprisingly, a functional enrichment analysis using the KEGG pathway database showed that the majority of the enriched pathways were indeed related to metabolic processes (Figure 2B). These results agree with our previous analyses. The network reconstruction of the enriched terms revealed that the pathways formed functional modules, as they clustered together and the word tag clouds of their annotations highlighted three major modules associated with glycolysis/gluconeogenesis, lipid metabolism, and TCA cycle (Figure 2C). Taken together, our results suggest that the vagus nerve is an important player in the control of liver metabolism.

### 2.3. Liver-Brain Axis Disruption or Cholinergic Signaling Impairment Result in a Metabolic Shift Towards Fatty Acid Biosynthesis in the Liver

**Figure 3:**
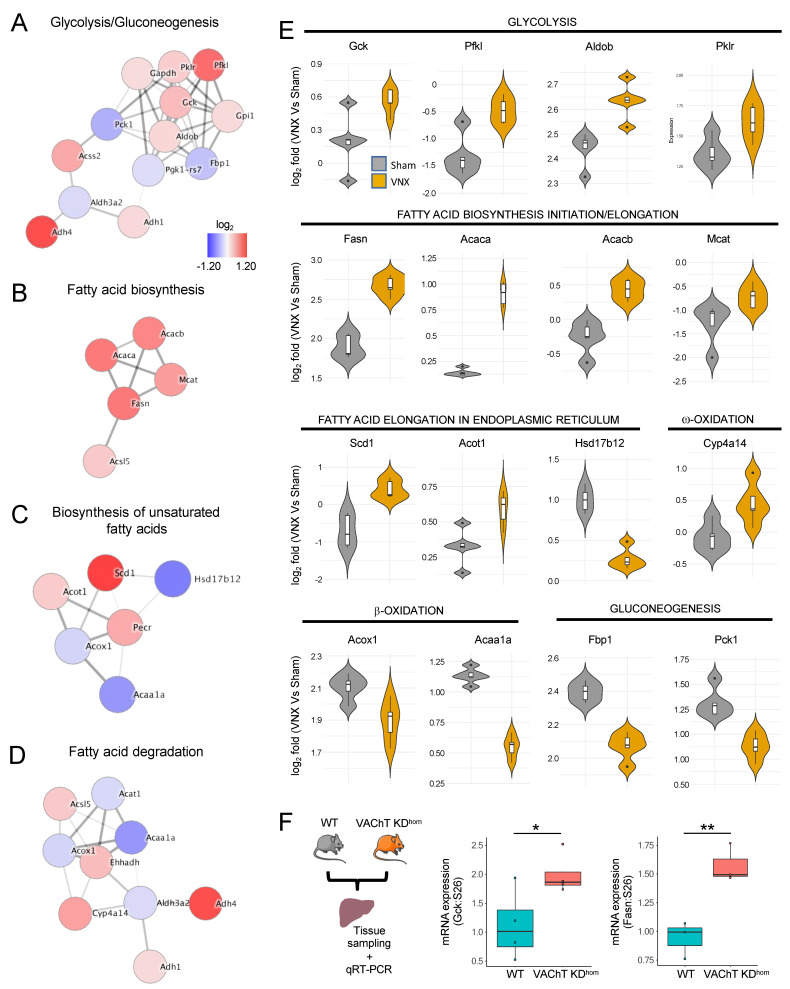
Liver metabolic shift following vagotomy. (**A**–**D**) Interaction network of differentially abundant proteins (*p* < 0.05, FDR adjusted-*p* < 0.05) within the selected enriched KEGG pathways. (**E**) Violin plots of the relative expression of the proteins grouped by physiological actions. (**F**) Relative *Gck* (n = 4/group) and *Fasn* (n = 3/group) expression in the livers of wild type (WT) and VAChT KD^hom^ mice. * *p* < 0.05; ** *p* < 0.01, student’s *t*-test.

We then reconstructed the metabolic framework of proteins within the pathways comprising the most represented functional modules: glycolysis/gluconeogenesis and lipid metabolism. The protein networks revealed that the vagus nerve has a broad regulatory effect, as it regulates the expression of multiple enzymes in each pathway, thus disrupting the balance of the global hepatic metabolic interactome (Figure 3A–D). When focusing the analyses on key enzymes that catalyze irreversible reactions (Figure 3E), we observed that vagotomy increased the expression of proteins involved in glycolysis (GCK, PFKL, ALDOB, PKLR), fatty acid biosynthesis initiation and elongation (FASN, ACACA, ACACB, MCAT), Fatty acid elongation in endoplasmic reticulum (SCD1 and ACOT1), as well as ω-oxidation (CYP4A14). Strikingly, the expression of key proteins involved in β-oxidation (ACOX1 and ACAA1A) and gluconeogenesis (FBP1 and PCK1) were reduced following VNX. Because acetylcholine is the main neurotransmitter in the parasympathetic vagal efferent fibers, we used the homozygous vesicular acetylcholine transporter knocked down mice (VAChT KD^hom^) to investigate the expression of key genes to glycolysis and fatty acid synthesis. In these animals, acetylcholine release is decreased due to a 60–70% reduction in VAChT expression [18]. Livers from VAChT KD^hom^ mice presented an increase in glucokinase (*Gck*) and Fatty Acid Synthase (*Fasn*) gene expression (Figure 3F). Together, these results point to a major alteration in the hepatic metabolic flux towards glycolysis and fatty acid biosynthesis mediated by the vagus nerve and vagal cholinergic system.

**Figure 4:**
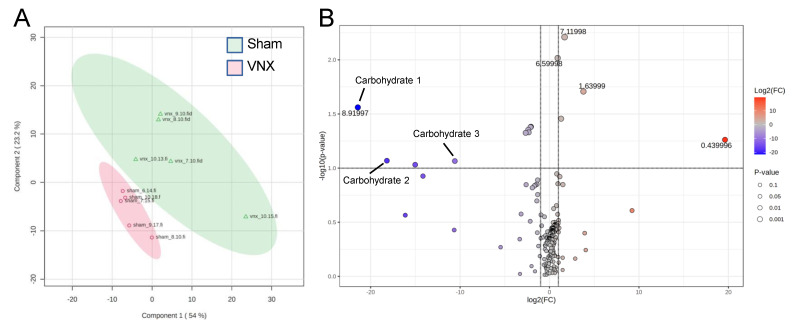
Liver metabolome remodeling following vagotomy. (**A**) PCA of metabolite abundance in VNX and Sham animals. (**B**) Volcano plot of metabolite cluster abundance. Blue and red colors respectively represent statistically decreased and increased expression in VNX mice compared to Sham animals (n = 5/group; *p* < 0.05, FDR adjusted-*p* < 0.05).

To validate the physiological relevance of these findings, we analyzed the metabolome of livers from sham and vagotomized animals. Vagotomy resulted in a distinct hepatic metabolomic profile when compared to the sham group (Figure 4A). Interestingly, the metabolite clusters composed of carbohydrates that were identified in the NMR spectra were reduced in the VNX group when compared to sham animals (Figure 4B). This corroborates the findings of the proteome analyses which pointed to increased glycolytic activity following vagotomy.

### 2.4. The Alterations in Hepatic Metabolic Machinery Are Independent of Major Changes in GI Tract Physiology

**Figure 5:**
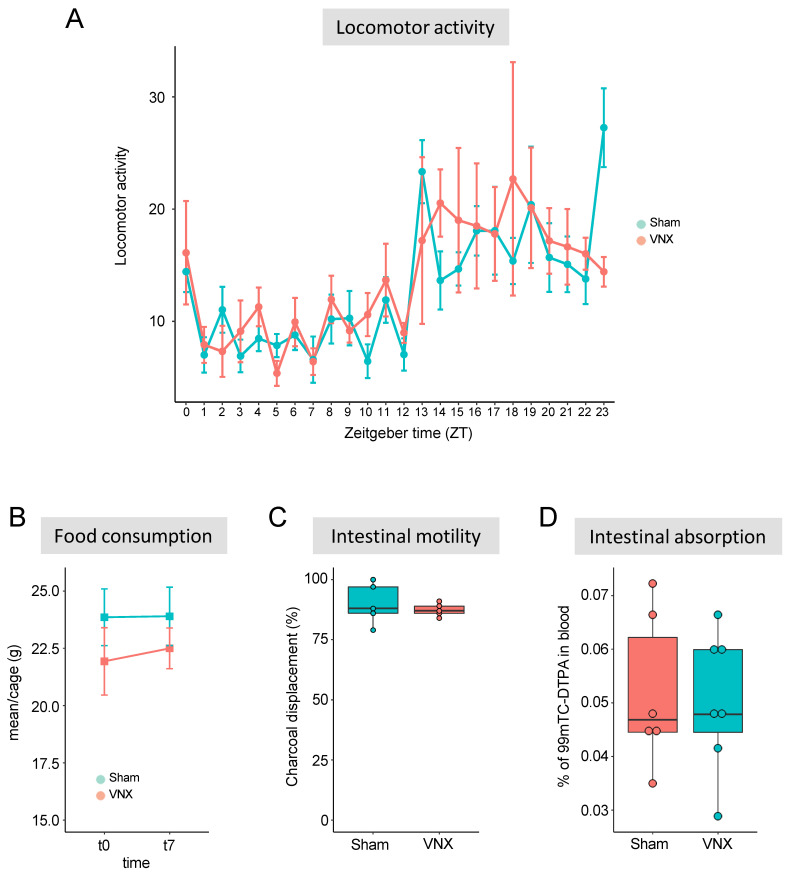
Physiological parameters following vagotomy. (**A**) Locomotor activity, (**B**) Food consumption, (**C**) Intestinal motility, and (**D**) Intestinal absorption. VNX = vagotomy; n = 5/group in each experiment.

The gastrointestinal tract is an important target of vagus nerve innervation. Therefore, we raised the possibility that the metabolic alterations in the liver shown in the proteomic analysis could be a consequence of changes in gastrointestinal physiology. To investigate this hypothesis, we first monitored the total locomotor activity of sham and VNX animals, using telemetry. We observed that animals of the two groups presented similar locomotor patterns throughout the day (Figure 5A) as well as food consumption before and after the surgery (Figure 5B), pointing to no major changes in feeding behavior. Furthermore, we did not observe significant differences in intestinal motility or intestinal absorption following vagotomy (Figure 5C,D). Our results suggest that the metabolic shift in mice submitted to VNX was not paralleled by major changes in nutrient handling in the gastrointestinal tract.

### 2.5. The Metabolic Shift Induced by Vagotomy Primes the Development and Severity of Metabolic Dysfunction-Associated Steatotic Liver Disease (MASLD)

Based on our findings that vagotomy shifts liver metabolism towards lipid accumulation by increasing glycolytic and fatty acid biosynthesis fluxes as well as reducing β-oxidation, we asked whether the disruption of vagus nerve circuitry would play a role in the development of MASLD.

**Figure 6:**
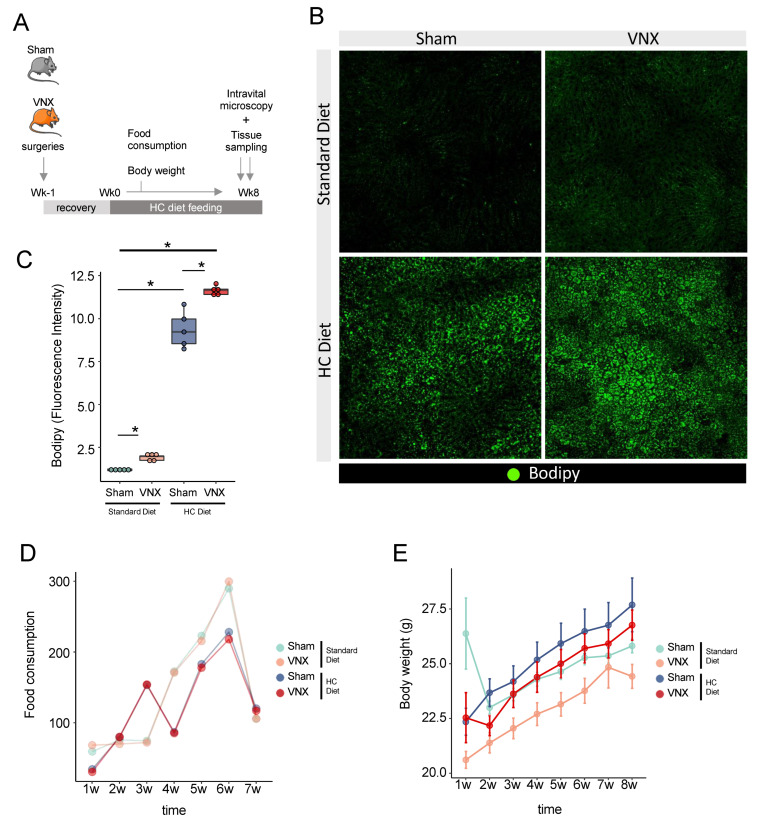
Vagotomy results in increased lipid accumulation in the liver following a high-carbohydrate (HC) dietary challenge. (**A**) Experimental design and protocol. (**B**) Representative in vivo confocal images of lipid accumulation assays using Bodipy. (**C**) Quantification of lipids in the liver of Sham and VNX mice (n = 5/group, * *p* < 0.05, One Way ANOVA with Newman–Keuls Multiple Comparison Test). (**D**,**E**) Food consumption (**D**) and Body weight (**E**) of mice throughout the experiment. VNX = vagotomy.

**Figure 7:**
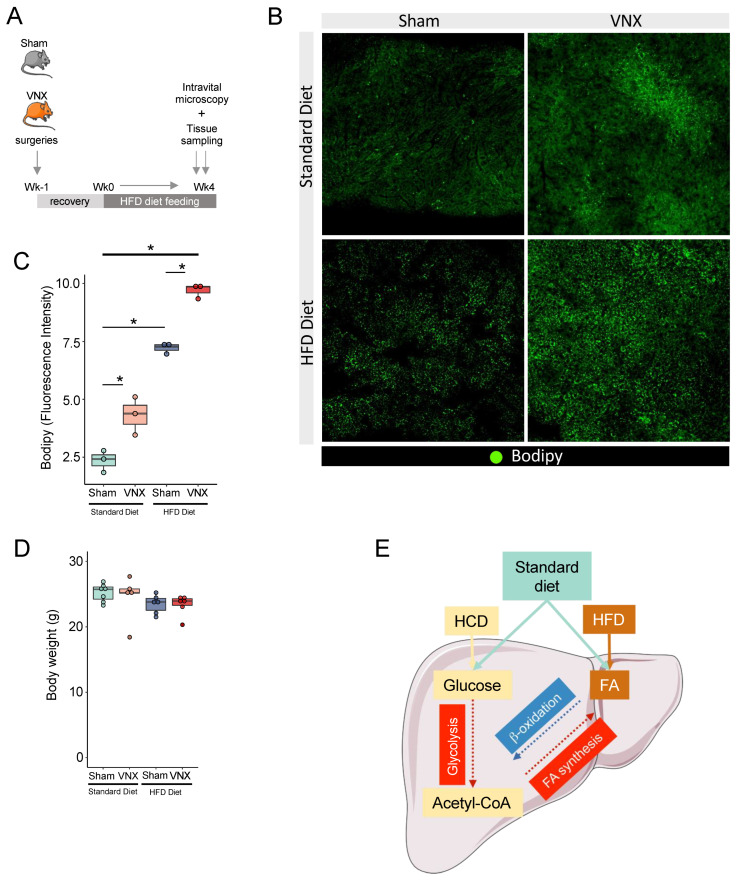
Vagotomy results in increased lipid accumulation in the liver following a high-fat (HFD) dietary challenge. (**A**) Experimental design. (**B**) Representative in vivo confocal images of lipid accumulation assays using Bodipy. (**C**) Quantification of lipids in the liver of Sham and VNX mice (n = 3/group, * *p* < 0.05, One Way ANOVA with Newman–Keuls Multiple Comparison Test). (**D**) Body weight of mice at the end of experiment. VNX = vagotomy. (**E**) Proposed model. In physiological conditions, the vagus nerve tunes carbohydrate and fatty acid metabolism to maintain metabolic homeostasis. When this circuitry is disrupted, the glycolytic and fatty acid synthesis processes are increased (red rectangles and arrows), whereas β-oxidation is reduced (blue rectangle and arrow). This creates a favorable metabolic flux that primes the liver to accumulate lipids. During a dietary challenge, such as high-carbohydrate diet (HCD) or high-fat diet (HFD), the high nutrient influx in the context of a metabolic shifted liver results in worsened hepatic steatosis.

For this purpose, we submitted Sham and VNX mice to a carbohydrate-rich dietary challenge (HC) and addressed lipid accumulation under physiological conditions by using high-definition intravital microscopy (Figure 6A). We found that HC-challenged mice presented a massive accumulation of lipid droplets within the hepatic parenchyma when compared to the group fed with a standard diet (Figure 6B,C). Vagotomy worsened liver lipid accumulation when compared to the Sham group, as observed by the increase in the parenchymal area occupied by lipid droplets. Surprisingly, vagotomy resulted in hepatic lipid accumulation even in standard diet-fed animals, corroborating and validating our proteomics data. Food consumption and body weight were not affected by any of the experimental procedures throughout the experiments (Figure 6D,E). We also performed a different dietary challenge using a high-fat diet (HFD) (Figure 7A) and observed similar results. HFD challenge resulted in liver lipid accumulation, which was more severe following VNX (Figure 7B,C). Importantly, the livers of mice submitted to vagotomy and fed a standard diet also displayed more lipid droplets when compared to the livers of the Sham group fed a similar diet. No alterations in body weight were detected (Figure 7D).

Taken together, our results show that the metabolic remodeling following vagotomy primes the liver to accumulate lipids, both under a standard diet feeding condition or during different dietary challenges.

## 3. Discussion

Our study identifies the vagus nerve as an important regulator of liver metabolic homeostasis, influencing key biochemical pathways involved in carbohydrate and lipid metabolism. Using a combination of quantitative proteomics and metabolomics, we identified key enzymes regulated by vagal signaling that control glycolysis/gluconeogenesis and fatty acid metabolism. Using VAChT KD^hom^ mice, a model with reduced acetylcholine release in vagal efferent fibers, we demonstrated an increase in glycolysis and fatty acid biosynthesis, along with a decrease in β-oxidation. Although the metabolic alterations were not accompanied by changes in food consumption, intestinal transit, or absorption, our in vivo imaging analysis revealed that vagotomized mice exhibited mild lipid accumulation in hepatocytes, even when maintained on a standard diet. This accumulation worsened following dietary challenges with high-carbohydrate or high-fat diets. Altogether, our findings provide a mechanistic framework linking vagal signaling in the liver to metabolic diseases such as MASLD and type II diabetes.

The liver is a central organ involved in carbohydrate and lipid metabolism, serving as a major energetic reservoir for other tissues [19] and the regulation of hepatic metabolic homeostasis is pivotal to whole-body metabolism. This balance is partially dependent on the sensing of metabolic states or nutrient availability and the release of neuroendocrine signals [20,21]. Most of these neural circuits are intrinsic to the vagus nerve, with vagal afferent fibers conveying sensory information as mechanical stretching, nutrients/metabolites and hormones, from gastrointestinal tract organs to the nucleus of the solitary tract (NTS) within the central nervous system [7,13,14,22,23,24,25]. The responses are then transmitted to the periphery through the efferent fibers of the nerve that originate predominantly from the dorsal motor nucleus of the vagus (DMN) and the ambiguous nucleus (NA) [22]. The brain-vagus-liver axis was shown to protect the liver against ectopic lipid accumulation by mediating the actions of leptin within the central nervous system, resulting in the activation of the dorsal vagal complex and increased triglyceride secretion by the liver [26]. These findings are in line with our main results, showing that the vagus nerve directly shapes liver metabolic function, especially carbohydrate and lipid metabolism. Importantly, this axis also prevented liver steatosis without changes in body weight, food intake, and circulating triglyceride levels [26], corroborating our findings. Recently, the importance of leptin signaling through the vagus nerve in preventing liver steatosis was also demonstrated in humans [27].

The vagus nerve neural circuit is a major component of cholinergic signaling, a pathway that has been associated with the control of metabolism. Acetylcholine, the main neurotransmitter released by efferent vagal fibers, exerts its functions by interacting with cholinergic receptors, including nicotinic and muscarinic receptors, both of which are expressed in the liver. In this manuscript, we observed that the disruption of the cholinergic system, as in VAChT KD^hom^ mice, resulted in overexpression of *Gck* and *Fasn*, key genes involved in glycolysis and fatty acid biosynthesis, respectively. In line with our findings, mice lacking the nicotinic acetylcholine receptor α7 subunit (α7nAChR^−/−^) showed more aggravated hepatic lipid accumulation and steatosis when fed a high-fat diet when compared with WT mice fed with the same diet. Remarkably, the pharmacological activation of α7nAChR in WT mice following a nutritional challenge partially reversed steatosis [28], an effect that involves the regulation of lipogenesis via the regulation of fatty acid synthase (*Fasn*) expression [29]. Independent studies also showed that impairment of cholinergic signaling mediated by α7nAChR worsened lipid accumulation within the liver in a diet-induced model of metabolic dysfunction-associated steatohepatitis (MASH) [30] or led to metabolic disorders [31]. Our data significantly expands this knowledge, as we show a complex alteration in the hepatic metabolic flux in the context of impaired cholinergic signaling, with the increase in the expression of glycolytic and fatty acid biosynthesis enzymes, concomitant with a decrease in key enzymes of the β-oxidation pathways (Figure 7E). The activation of the cholinergic signaling in the liver through the cholinergic receptor nicotinic alpha 2 subunit (Chrna2) induced the metabolic adaptations to caloric overload and protective mechanisms against steatosis [32]. In this study, it was demonstrated that the hepatic population of resident macrophages (Kupffer cells) plays a crucial role in initiating this signaling, as they serve as a significant source of acetylcholine. Interestingly, we have previously shown that vagus nerve activation increases the expression of choline O-acetyltransferase (*Chat*), a key enzyme for acetylcholine synthesis, in this macrophage population [33]. Altogether, these findings suggest that liver metabolism may be tightly regulated by a synergistic cholinergic interaction between the vagus nerve, as reported in this study, and resident macrophages.

Chronic high-calorie ingestion reduces vagal excitability and decreases signaling to the central nervous system [34,35]. Such disruption is sufficient to promote overconsumption of food and weight gain [36,37] and, as described here, likely leads to alteration in the metabolic protein network within the liver. Altogether, these findings suggest vagus nerve-mediated signaling disruption as a putative important event for the onset of metabolic disorders. Within the spectrum of metabolic diseases, metabolic dysfunction-associated steatotic liver disease (MASLD) is recognized as a major health burden worldwide [38]. MASLD is a condition characterized by the initial accumulation of fat in the liver that can progress to inflammation and fibrosis (MASH) and that is the fastest-growing indication for liver transplantation [39]. Interestingly, the frequency of de novo MASLD ranges between 18% and 30% in patients submitted to liver transplant [39,40,41], and as in the general population, it occurs in the context of altered metabolism, suggesting the major relevance of host factors [42]. Because reinnervation of hepatic parenchyma is limited after transplantation [43], our findings may provide new insights into this condition as well.

In addition to the direct effect of vagal signaling on liver parenchyma, it is important to consider the microbiota and its products, which may interact with gut-brain neural circuits or the gut-liver axis. In this context, the vagus nerve expresses receptors for certain microbiota-derived peptides and metabolites, including lipopolysaccharides (LPS) and short-chain fatty acids (SCFAs), which are produced through the fermentation of dietary fibers [44]. Both LPS and SCFAs can directly modulate the activation of vagal afferent fibers [45,46,47]. Additionally, SCFAs are absorbed by the gut epithelium and reach the liver via the portal circulation, where they serve as anabolic substrates for the hepatic parenchyma. For example, propionate is utilized in gluconeogenesis, whereas acetate and butyrate contribute to lipogenesis [48]. LPS is also absorbed in the intestines and in the liver, it may regulate lipogenesis through the toll-like receptor 4 [49]. Therefore, the profound metabolic effects observed following the disruption of vagus nerve-mediated neural circuits may, at least in part, result from alterations in the signaling tone of microbiota-derived metabolites through this nerve or from changes in the metabolism of these products in the context of the metabolic shift induced by vagotomy.

In conclusion, this study describes the liver-brain axis mediated by the vagus nerve as an important regulator of the hepatic metabolic landscape. We found that the disruption of this neural circuit leads to a metabolic shift in the liver towards fatty acid biosynthesis, which primes the organ to accumulate lipids.

## 4. Materials and Methods

### 4.1. Ethical Approval

All experimental procedures were performed in accordance with protocols approved by the Institutional Animal Use Committee and in compliance with the Brazilian legislation of animal care and experimentation. The number of animals was based on previous studies [33,50].

### 4.2. Mouse Models and Surgical Procedure

Male C57/Bl6J mice (8–10 weeks old) used in this study were obtained from CEBIO-UFMG. Dr. Marco Antônio Prado, from the Robarts Research Institute (University of Western Ontario, London, ON, Canada) kindly provided the VAChT KD^hom^ and WT mice used in the experiments (background C57/Bl6J-A—agouti). Animals were housed under a 12-h light/dark cycle at 25 °C, with unrestricted access to food and water. All the experiments were performed mid-through the light phase at ZT6 ± 30 min (ZT; Zeigtgeber Time) as the circadian light cycle influences the majority of metabolic pathways in the murine liver [51]. Animals were randomly divided into the experimental groups.

Vagotomy (VNX) was performed in mice anesthetized with ketamine/xylazine (i.p.; 80 mg/kg and 15 mg/kg, respectively). After a midline incision in the cervical region, the left cervical trunk of the vagus nerve was exposed, ligated with a 4–0 silk and sectioned [33]. Sham mice were submitted to similar procedures without nerve transection. All animals were allowed to recover for 7 days before other experiments.

### 4.3. Proteomics

To obtain an overview of the proteins expressed in our phenotype, we performed the proteomics assay, which also served as the basis for understanding possible subsequent physiological changes in our model. After seven days of recovery from surgery, we collected the livers (n = 5 animals/group). Samples were washed with physiological saline, frozen in liquid nitrogen, and later stored in a freezer at −80 °C.

#### 4.3.1. Liver Tissue Preparation

Tissues were snap-frozen in liquid nitrogen and grounded to powder in a mortar chilled with liquid nitrogen using a pestle. Tissues were stored at −80 °C until further use. About 6 milligrams (mg) of sample was resuspended in 100 µL of lysis buffer containing 6 M Urea, 2 M Thiourea, 40 mM 2-chlorocetamide, 20 mM TEAB, 100 mM TCEP and protease and phosphatase inhibitors. The samples were sonicated at 40 Hz, 3× for 15 s in ice-cold for DNA fragmentation. Then, the samples were incubated at 28 °C and 650 rpm for 2 h for proper protein lysis, reduction, and alkylation. After that, protein quantification was quantified using Qubit assay (Thermo Fisher Scientific, Waltham, MA, USA) and 250 μg of proteins of each sample were collected for protein digestion. Prior to digestion, samples were diluted with TEAB 50 mM to reach 0.6 M urea and pH 8. Trypsin was added at 1:50 (*w*/*w*) (enzyme-to-protein ratio) and protein digestion was carried out at 25 °C for 16 h. The enzymatic reaction was quenched with TFA 1% (*v*/*v*) (final concentration). Samples were desalted using self-made micro-columns packed with OLIGO R2 and R3 Reversed-Phase Resin (Thermo Fisher Scientific, Waltham, MA, USA). Desalted peptides were dried in a SpeedVac and stored at −20 °C until further use.

#### 4.3.2. Peptide Isotopic Labeling

Peptides were labeled according to the “On-column” dimethyl labeling protocol [52], with minor modifications. Briefly, the peptides were reconstituted in 1 mL of 5% (*v*/*v*) formic acid (CH_2_O_2_). SepPak columns (Waters, Milford, MA, USA) were: (i) activated with 4 mL of 100% ACN; (ii) conditioned with 4 mL of solution A (0.6% acetic acid (*v*/*v*)); (iii) loaded with the samples; (iv) washed with 4 mL of solution A; (v) incubated with the respective labeling reagent for 30 min. Sham and VNX groups were labeled with different labeling reagents (Sham = light and VNX = medium). The isotopic labeling reagents contained 500 µL of 50 mM sodium-phosphate buffer (pH 7.5), 250 µL of 0.6 M sodium cyanoborohydride (NaBH_3_CN) and 250 µL of a 4% (*v*/*v*) solution containing the respective formaldehyde: CH_2_O (light) or CD_2_O (medium); (vi) washed with 4 mL of solution A; (vii) eluted with an increasing gradient (20%, 50%, 80% and 100% (*v*/*v*) ACN + 0.6% (*v*/*v*) acetic acid). Samples were dried in a SpeedVac and reconstituted in 0.1% TFA (*v*/*v*) and quantified using Qubit (Thermo Fisher Scientific, Waltham, MA, USA). The labeling efficiency was checked using the MALDI-TOF mass spectrometer (Autoflex III, Bruker, Billerica, MA, USA) with the m/z range of 780–2500 Da. Then, the samples from the light and medium groups were combined in the same tube with the same ratio of 1:1 labeled peptides (Sham:VNX). Finally, samples were dried using a SpeedVac and stored at −20 °C.

#### 4.3.3. Mass Spectrometry Analysis

Fractions were resuspended in 0.1% (*v*/*v*) formic acid (solvent A) and the peptides were separated and analyzed using an EASY-nLC system (Thermo Fisher Scientific, Waltham, MA, USA) with a two-column system setup coupled to a Q-Exactive HF mass spectrometer (Thermo Fisher Scientific, Waltham, MA, USA). The pre-column was 3 cm in length × 100 µm i.d. and was packed with 5 μm particles (Reprosil-Pur C18-AQ, Dr. Maisch GmbH, Ammerbuch, Germany). The analytical column was 18 cm in length × 75 µm i.d. and was packed with 3 μm particles (Reprosil-Pur C18-AQ, Dr. Maisch GmbH, Ammerbuch, Germany). For the peptide separation, the following chromatographic gradient was used: 1–3% solvent B (95% (*v*/*v*) acetonitrile + 0.1% (*v*/*v*) formic acid) in 0–3 min; 3–28% solvent B in 45 min; 28–45% solvent B in 10 min; 45–95% solvent B in 3 min at 250 nL/minute. For the MS analysis, the instrument was operated in positive polarity and DDA mode. The eluted peptide ions were resolved (MS1 mass range = m/z 400–1400) at 120,000 resolution (at m/z 200). The MS1 AGC target settings were set to allow accumulation of up to 3 × 10^6^ charges for up to 120 ms injection time. The 20 most intense peptide ions (Top 20) were selected with an isolation window of 1.2 Th and the normalized collision energy for HCD fragmentation was set to 32. For MS2 AGC target settings were set to allow accumulation of up to 10^5^ ions for up to 120 ms. The fragment ions were resolved with 15,000 resolution (at m/z 200) and selected precursor ions were excluded. Spectra files (.raw) were viewed in Xcalibur v3.0 (Thermo Fisher Scientific, Waltham, MA, USA).

### 4.4. Proteomics Data Analysis

The raw spectra (.raw) were analyzed with MaxQuant 1.5.8.3. Spectra were searched against the SwissProt *Mus musculus* FASTA database (downloaded October 2017) using the Andromeda search engine [53]. Search parameters included a 20 ppm tolerance for the first search and a 4.5 ppm tolerance for the main search. Dimethyl labeling was set using the following modifications: “Dimethyl-Lys0” and “Dimethyl-Nterm0” for light labeling, and “Dimethyl-Lys4” and “Dimethyl-Nterm4” for medium labeling. Trypsin was chosen as the enzyme with a max of two missed cleavage sites. Carbamidomethyl of cysteine was set as a fixed modification and oxidation of methionine and acetylation of protein N-terminus were set as variable modifications. “Match between runs” was enabled with a matching time window of 0.7 min and an alignment time window of 20 min. Peptide identification required at least 1 unique peptide and was filtered to 0.01 false discovery rate (FDR).

Protein intensity was log2-transformed and then normalized by the column median and row mean. Reverse and contaminant proteins were removed using Perseus software (version 2.0.9.0). Statistical analysis one-way ANOVA (*p*-value < 0.05) was applied to find differentially regulated features with FDR 0.05 strategy to adjust the *p*-values using DanteR. To explore the data within the Kegg database context, we used Functre2 [54]. Kegg functional enrichment and further data visualization were performed in R (version 4.1.3) and the following R-packages: ClusterProfiler [55], EnhancedVolcano, and pheatmap. Protein network analysis was performed using String and ClueGo [56,57] for Cytoscape (version 3.7.2).

### 4.5. Metabolomics

250 mg liver samples were homogenized in a 1:5 cold water:methanol solution (total of 485 µL). Then a solution of cold chloroform:water at 1:1.1 (total of 840 µL) was added to each homogenate. The samples were then vortexed for 1 min, followed by a 10 min incubation in ice on an orbital shaker, and finally centrifuged for 10 min at 2000× *g*, 4 °C. The supernatants containing the polar fractions were separated for nuclear magnetic resonance (NMR) analysis.

A total of 250 μL of the extracted sample was diluted 1:1 in a sodium phosphate buffer with a pH of 7.4. The mixture was carefully homogenized and 600 μL (20 mM final buffer concentration) was transferred to a 5 mm NMR tube for analysis and 10% deuterium water (D_2_O) was added. The 1H NMR spectra were analyzed using a Bruker Avance Neo 600 MHz spectrometer equipped with a PABBO 5 mm SmartProbe (Bruker™ Biospin, Rheinstetten, Germany). 1H NMR spectra were acquired using a *noesygppr1d* (Bruker^®^) with presaturation during the relaxation delay and mixing time and spoil gradient at 298 K, 64 K fid size, 1024 scans, a spectral width of 20 ppm, a transmitter frequency offset of 4.70 ppm, 2.6 s acquisition time and 7 s relaxation delay. 1H-1H total correlation spectroscopy (*dipsi2esgpph*) and 13C-1H HSQC (*hsqcetgpprsisp2.3*) experiments were also performed for the assignments. After the acquisition, the spectra were uploaded to MestReNova 14.2.1–27684 on the NMRBox platform [58] to be processed with 128 K fd size, phase, and baseline corrected and calibrated. Finally, the binning table was created using the average SUM method with a bucket size of 0.02 ppm, and the data were normalized to the total sum of peak intensities. The water region was deleted for the analysis. The binning table was imported into R and analyzed using the metaboanalystR package [59]. Pre-processing of the data included log transformation, Pareto scaling, and normalization to correct systematic deviations. Data integrity checks were performed to identify and handle missing values or outliers. PLS-DA was used to maximize the separation between the sham and SNX groups. 2D score plots of the first two latent variables (LV1 and LV2) were created using the PlotPLS2DScore function to visualize the separation of the samples. Important characteristics that contributed to the separation of the groups were identified using Variable Importance in Projection (VIP) scores and visualized using the PlotPLSImp function. Differential metabolite analysis was performed using the Volcano. Anal function with the following parameters: Fold change threshold: 2.0 Adjusted *p*-value threshold: 0.1 FDR correction: applied Volcano plots were created using the PlotVolcano function to identify significant features based on log2 fold change and adjusted *p*-values. Statistical significance for the differential analysis was set at an adjusted *p*-value < 0.05. In addition, the COLMARm database was used, a platform to verify the existence of compounds already quantified in NMR. This database is divided into four steps in which you can check the processing of spectra, select peaks and peak fitting and perform spectral referencing by selecting solvents and compounds [60,61].

### 4.6. qRT-PCR

Total mRNA was extracted by using the Aurum total RNA mini kit and reverse transcribed by using iScript cDNA synthesis kit (BioRad Laboratories, Hercules, CA, USA). qRT-PCR reactions were performed using the iTaq Universal SYBR Green Supermix (BioRad Laboratories, Hercules, CA, USA). Each transcript level was normalized to the S26 gene. WT group was used as a references for data normalization in WT versus VAChT KD^hom^ comparison. Data were analyzed by the 2^−ΔΔCt^ method. The primers used were as follows:

Mitochondrial ribosomal protein S26 (*S26*; Gene ID: 99045)
Forward: CGATTCCTGACAACCTTGCTA
Reverse: CGTGCTTCCCAAGCTCTATGT
Fatty Acid Synthase (*Fasn*; Gene ID: 14104)
Forward: GGAGGTGGTGATAGCCGGTAT
Reverse: TGGGTAATCCATAGAGCCCAG
Glucokinase (*Gck*; Gene ID: 103988)
Forward: TGAGCCGGATGCAGAAGGA
Reverse: GCAACATCTTTACACTGGCCT

### 4.7. Locomotor Activity

A telemetry sensor was surgically placed in the abdominal cavity of VNX and control animals (n = 4 animals/group). The locomotor activity was telemetrically monitored through the signal emitted by the capsule implanted in the abdominal cavity and received by the signal receiver under the experimental vats connected to an acquisition software. Data was acquired in continuous mode and subsequently processed. Telemetry records were made for 14 days.

### 4.8. Intestinal Motility

VNX or Sham animals (n = 5 animals/group) received, by gavage, 300 μL of activated carbon solution (10% *v*/*v* of activated carbon:5% *v*/*v* of gum arabic), at ZT6 ± 30 min. Motility was determined in isolated intestines 20 min after gavage by measuring the distance traveled by the activated carbon solution and dividing it by the value of the total length of the intestine. The result was expressed as a percentage of the total length of the small intestine.

### 4.9. Intestinal Absorption

All animals (Sham: n = 6 animals/group; VNX: n = 7/group) received by gavage 100 μL of solution containing diethyleneaminopentacetic acid labeled with 18.5 MBq 99mTechnetium (99mTc-DTPA). Four hours after gavage, blood samples were collected and submitted to radiation determination (cpm) in a gamma radiation counter (Wallac Wizard Gamma counter, PerkinElmer, Shelton, CT, USA). The results obtained were compared with the standard of 99mTc-DTPA and calculated as the percentage product of blood cpm times 100 cpm of administered dose.

### 4.10. Dietary Challenges

To analyze the temporal evolution of diet-induced metabolic changes, we introduced a high-refined carbohydrate (HC) or high-fat (HFD) diet in vagotomized animals and their control.

The macronutrient composition of the HC diet (4.4 kcal/g) was 74.2% carbohydrate, 5.8% fat, and 20% protein. It is important to note that the HC diet contains at least 30% refined sugars, mainly sucrose. The standard diet (4.0 kcal/g) composition was 65.8% carbohydrate, 3.1% fat, and 31.1% protein (Nuvilab, Quimtia, Colombo, PR, Brazil). The animals (n = 5 animals/group) were fed standard laboratory chow or experimental diet for a period of 8 weeks after vagotomy.

In the HFD diet model, mice were fed standard laboratory chow or high-fat chow for a period of 4 weeks after vagotomy. High-fat diet (7.0 kcal/g) was composed of 24.5% carbohydrate, 61.1% fat, and 14.5% protein.

At the end of the dietary treatment, animals were euthanized. Liver samples were frozen in liquid nitrogen and stored at −80 °C until processing.

### 4.11. Liver Intravital Microscopy

The intravital confocal microscopy images were performed as described [33,50]. In summary, mice from the Sham and VNX groups were anesthetized with an intraperitoneal injection of ketamine (80 mg/kg) and xylazine (15 mg/kg), at the time of ZT6. A laparotomy was performed to expose the liver and then the left lobe was collected and placed in a petri dish containing saline. To visualize the lipid droplets in vivo, the Bodipy dye (1.5 μg/mouse diluted in DMSO, Thermo Fisher Scientific, Waltham, MA, USA) was placed directly in the left lobe of the liver collected after surgery. Images were obtained using a Nikon A1R confocal microscope (Nikon Instruments, Melville, NY, USA). Images were acquired with a 20× Plan Apo objective and analyzed using the ImageJ software (version 1.54f).

### 4.12. Statistical Analysis

Differences between the two samples were analyzed for significance using an unpaired two-tailed Student’s *t*-test. Differences between three or more groups were analyzed using One-way ANOVA with Newman–Keuls Multiple Comparison Test. Values are from multiple biological replicates within an experiment and are reported as the mean ± standard deviation and plots that highlight the distribution of individual data. Statistical significance was set at *p* < 0.05.

## Data Availability

The mass spectrometry proteomics data have been deposited to the ProteomeXchange Consortium via the PRIDE [62] partner repository with the dataset identifier PXD041052.

## Supplementary Materials

The following supporting information can be downloaded at: https://www.mdpi.com/article/10.3390/ijms26052166/s1.
